# ﻿Unveiling Sordariomycetes taxa associated with woody litter in Northern Thailand

**DOI:** 10.3897/mycokeys.115.145330

**Published:** 2025-03-17

**Authors:** Chayanard Phukhamsakda, Kevin D. Hyde, Milan C. Samarakoon, Johnny Louangphan, Kedsara Navasit, Fatimah Al-Otibi, Chitrabhanu S. Bhunjun

**Affiliations:** 1 Center of Excellence in Fungal Research, Mae Fah Luang University, Chiang Rai 57100, Thailand; 2 CAS Key Laboratory for Plant Diversity and Biogeography of East Asia, Kunming Institute of Botany, Chinese Academy of Science, Kunming, Yunnan 650201, China; 3 Department of Botany and Microbiology, College of Science, King Saud University, P.O. Box 22452, Riyadh 11495, Saudi Arabia; 4 Department of Entomology and Plant Pathology, Faculty of Agriculture, Chiang Mai University, Chiang Mai 50200, Thailand; 5 School of Science, Mae Fah Luang University, Chiang Rai 57100, Thailand

**Keywords:** 2 new records, 2 new taxa, anthostomella-like taxa, phylogeny, saprobes, taxonomy

## Abstract

Sordariomycetes species are abundant in woody litter samples. In this study, we introduce two novel species, *Diaporthethailandica* (Diaporthaceae) and *Occultithecachiangraiensis* (Xylariaceae), from woody litter materials. We also describe a new host record of *D.tulliensis* and a new geographical record for *D.melonis.* All collections were identified based on morphology and phylogenetic analyses of combined datasets. The morphologies of the taxa fit the generic concepts of *Diaporthe* and *Occultitheca*, respectively. *Diaporthethailandica* formed a sister clade with *D.raonikayaporum* but differs from *D.raonikayaporum* in the sizes of conidiomata, conidiogenous cells, and beta conidia. *Diaporthethailandica* also differs from *D.raonikayaporum* by the absence of gamma conidia. *Occultithecachiangraiensis* differs from the sister taxon *O.rosae* in having smaller ascomata and a thicker mucilaginous sheath. We also provide a synopsis of *Occultitheca* species with details on their morphology, host, and country. These findings provide valuable insights into the diversity and ecological roles of Sordariomycetes, emphasising the need for continued exploration of fungal biodiversity in various environments.

## ﻿Introduction

Plant litter plays an important role in shaping ecological processes and supporting biodiversity, which represents a major source of organic carbon and nutrient cycling (Zhang et al. 2023). As natural decomposers, fungi hold a significant role in the breakdown of woody litter by degrading complex organic compounds efficiently (Dashtban et al. 2010; Hyde et al. 2019; Mapook et al. 2022). Although woody litter harbours various groups of fungi, comprehensive studies are limited (Hyde et al. 2020a; Phukhamsakda et al. 2022; Wanasinghe and Mortimer 2022; Xu et al. 2022; Madagammana et al. 2023).

*Diaporthe* (Diaporthaceae, Diaporthales, Sordariomycetes, Ascomycota) was established by Fuckel (1867) with *D.alnea* as the type species. *Diaporthe* is an important plant pathogen that also comprises endophytes and saprobes on a wide range of hosts (Dissanayake et al. 2017; Hyde et al. 2020b; Phukhamsakda et al. 2020; Bhunjun et al. 2022). As pathogens, *Diaporthe* cause diebacks, cankers, leaf spots, blights, melanoses, stem-end rots, and gummosis on economically and ecologically important plants (Gomes et al. 2013; Manawasinghe et al. 2021; Bhunjun et al. 2024a). The sexual morph of *Diaporthe* is characterised by immersed ascomata, erumpent pseudostroma with elongated perithecial necks, and unitunicate asci that produce hyaline ascospores (Udayanga et al. 2011; Hongsanan et al. 2023). The asexual morph has ostiolate conidiomata, cylindrical phialides, and aseptate and hyaline conidia (alpha, beta, and gamma) (Udayanga et al. 2011; Gomes et al. 2013). Molecular approaches are essential for accurate identification due to overlapping morphological characters among distinct species (Bhunjun et al. 2022; Norphanphoun et al. 2022). The taxonomy of *Diaporthe* species has been the subject of several studies (Gomes et al. 2013; Udayanga et al. 2014; Gao et al. 2017; Sun et al. 2021; Norphanphoun et al. 2022). The genus has recently been revised, and 31 species were synonymised based on multi-gene phylogeny, GCPSR (Genealogical Concordance Phylogenetic Species Recognition), as well as coalescence-based analyses of ITS, *tef1*, *tub2*, *cal*, and *his3* sequences (Dissanayake et al. 2024). Pereira and Phillips (2024) further reduced 53 species to synonymy and introduced a new species, *D.pygmaeae*, based on several molecular approaches.

*Occultitheca* (Xylariaceae, Xylariales) is characterised by immersed ascomata, short pedicellate asci with J+ apical ring, brown ascospores with hyaline dwarf cells, and a straight germ slit (Rogers and Ju 2003; Samarakoon et al. 2022). The genus is notable for having the uppermost ascospore distant from the ascus apex (Rogers and Ju 2003; Samarakoon et al. 2022). The second species, *O.rosae*, was described almost two decades later from a dead branch of *Rosa* species in Guizhou, China (Samarakoon et al. 2022). Tian et al. (2024) introduced *O.ananasi* from dead pineapple leaves from Chiang Rai, Thailand, based on morphological and molecular analyses.

Chiang Rai and Chiang Mai are located in the northern part of Thailand and are considered biodiversity hotspots (Hyde et al. 2018). During the study of woody litter microfungi in northern Thailand, two novel species and two new records were discovered based on morphology and multigene phylogeny. This underscores a critical gap in our understanding of fungal biodiversity and its ecological significance in the area, thus highlighting the need for further research (Hyde et al. 2024).

## ﻿Materials and methods

### ﻿Fungal collection, isolation, and observation

Woody litter samples were collected from forest areas in Chiang Mai and Chiang Rai Provinces, Thailand. The area is covered with a canopy of tall trees, such as dipterocarp species and *Bambusa* species. The specimens were maintained in paper bags for transport to the laboratory. The fungal structures were observed using a Leica EZ4 stereomicroscope (Leica Microsystems (SEA) Pte Ltd, Singapore). and photographed using a Nikon ECLIPSE Ni compound microscope (Nikon, Japan) equipped with a Nikon DS-Ri2 camera. Tarosoft (R) Image Frame Work version 3.9.3.74 was used for measurements, and Adobe Photoshop CS6 software was used for the photo plates.

Single spore isolation was conducted to obtain pure culture on potato dextrose agar (PDA) as described in Senanayake et al. (2020). The culture plates were incubated at room temperature (25 ± 5 °C) for 4 weeks. Herbarium materials were deposited in the Mae Fah Luang University Fungarium (MFLU), and ex-type living cultures were deposited in the Mae Fah Luang University Culture Collection (MFLUCC) in Chiang Rai, Thailand. Index Fungorum (Index Fungorum 2024) and Facesoffungi numbers (FoF) (Jayasiri et al. 2015) were obtained. The data of the novel species will also be uploaded to the Fungalpedia website (Hyde et al. 2023).

### ﻿DNA extraction, amplification, and sequencing

Fresh mycelium was scraped from a 4-week-old culture on PDA, and DNA was extracted using the E.Z.N.A. Forensic DNA Kit (BIO-TEK) according to the manufacturer’s instructions. The polymerase chain reaction (PCR) was used to amplify the ITS region (ITS5/ITS4) (White et al. 1990), *cal* (CAL228F/CAL737R) (Carbone and Kohn 1999), *his3* (CYLH3F/H3-1b) (Glass and Donaldson 1995), LSU (LR0R/LR5) (Vilgalys and Hester 1990), *rpb2* (fRPB25f/fRPB2-7cR) (Liu et al. 1999), *tef1* (EF-728F/EF-986R) (Carbone and Kohn 1999), and *tub2* (T1/T22 and BT2a/BT2b) genes (Glass and Donaldson 1995; O’Donnell and Cigelnik 1997). The PCR conditions for each primer were set up following Samarakoon et al. (2022) and Dissanayake et al. (2024). A PCR reaction was carried out in a 25 μL reaction volume containing 12.5 µL of 2 × PCR MasterMix, 9.5 µL of double-distilled water, 1 µL of 20 µmol for each forward and reverse primer, and 1 µL of 30 ng of DNA template. The PCR products were visualised on 1% agarose gels with 6 μl of 4S green dye per 100 mL. Successful PCR products were purified and sequenced by Biogenomed Co., Ltd., South Korea. The newly generated sequences were deposited in GenBank (Tables 1, 2).

**Table 1.** GenBank accession numbers of the taxa used in the phylogenetic analyses of Diaporthesectionsojae.

Species	Strain	ITS	*tef1*	*tub2*	*cal*	*his3*
*Diaportheacaciarum*	CBS 138862^T^	KP004460	N/A	KP004509	N/A	KP004504
*D.amaranthophila*	MAFF 246900^T^	LC459575	LC459577	LC459579	LC459583	LC459581
*D.amaranthophila*	MAFF 246901	LC459576	LC459578	LC459580	LC459584	LC459582
*D.ambigua*	CBS 114015^T^	MH862953	KC343736	KC343978	KC343252	KC343494
*D.ambigua*	CBS 117167	KC343011	KC343737	KC343979	KC343253	KC343495
*D.angelicae*	CBS 111592^T^	KC343027	KC343753	KC343995	KC343269	KC343511
*D.angelicae*	CBS 100871	KC343025	KC343751	KC343993	KC343267	KC343509
*D.arctii*	CBS 139280^T^	KJ590736	KJ590776	KJ610891	KJ612133	KJ659218
*D.arezzoensis*	MFLU 19-2880^T^	MT185503	MT454019	MT454055	N/A	N/A
*D.batatas*	CBS 122.21^T^	KC343040	KC343766	KC344008	KC343282	KC343524
*D.beilharziae*	BRIP 54792^T^	JX862529	JX862535	KF170921	N/A	N/A
*D.biguttulata*	CFCC 52584^T^	MH121519	MH121561	MH121598	MH121437	MH121477
*D.biguttulata*	CFCC 52585	MH121520	MH121562	MH121599	MH121438	MH121478
*D.brasiliensis*	CBS 133183^T^	KC343042	KC343768	KC344010	KC343284	KC343526
*D.brasiliensis*	LGMF926	KC343043	KC343769	KC344011	KC343285	KC343527
*D.breyniae*	CBS 148910^T^	ON400846	ON409188	ON409186	ON409189	ON409187
*D.caatingaensis*	URM7485^T^	KY085927	KY115604	KY115601	KY115598	KY115605
*D.caatingaensis*	URM7484	KY085928	N/A	KY115602	KY115599	KY115606
*D.caryae*	CFCC 52563^T^	MH121498	MH121540	MH121580	MH121422	MH121458
*D.caryae*	CFCC 52564	MH121499	MH121541	MH121581	MH121423	MH121459
*D.chimonanthi*	SCHM 3614^T^	AY622993	N/A	N/A	N/A	N/A
*D.chimonanthi*	SCHM 3603	AY620820	N/A	N/A	N/A	N/A
*D.cichorii*	MFLUCC 17-1023^T^	KY964220	KY964176	KY964104	KY964133	N/A
*D.cinnamomi*	CFCC 52569^T^	MH121504	MH121546	MH121586	N/A	MH121464
*D.cinnamomi*	CFCC 52570	MH121505	MH121547	MH121587	N/A	MH121465
*D.citriasiana*	ZJUD30^T^	JQ954645	JQ954663	KC357459	KC357491	N/A
*D.citriasiana*	ZJUD81	KJ490616	KJ490495	KJ490437	N/A	KJ490558
*D.convolvuli*	CBS 124654^T^	KC343054	KC343780	KC344022	KC343296	KC343538
*D.convolvuli*	FAU649	KJ590721	KJ590765	N/A	KJ612130	KJ659210
*D.coracoralinae*	URM 8912^T^	PP192078	PP430449	PP402241	PP408214	PP421133
*D.coracoralinae*	FCCUFG 39	PP192079	N/A	PP402242	PP408215	PP421134
*D.cucurbitae*	DAOM 42078^T^	KM453210	KM453211	KP118848	N/A	KM453212
*D.cucurbitae*	CBS 136.25	KC343031	KC343757	KC343999	KC343273	KC343515
*D.cuppatea*	CBS 117499^T^	KC343057	KC343783	KC344025	KC343299	KC343541
*D.cyatheae*	YMJ-1364^T^	JX570889	KC465406	KC465403	KC465410	N/A
*D.discoidispora*	ZJUD89^T^	KJ490624	KJ490503	KJ490445	N/A	KJ490566
*D.discoidispora*	GZCC 22-0065	OP056659	OP150498	OP150576	OP150655	OP150730
*D.eleutharrhenae*	01^T*^	OK017069	OK017070	OK017071	N/A	N/A
*D.eleutharrhenae*	02*	OK648457	OK648458	OK648459	N/A	N/A
*D.fici-septicae*	NCYUCC 19-0108^T^	MW114349	MW192212	MW148269	N/A	N/A
*D.fici-septicae*	MFLU 20-20178	MW114348	MW192211	MW148268	N/A	N/A
*D.foliorum*	CMRP 1321^T^	MT576688	MT584310	MT584327	MT584341	MT584338
*D.foliorum*	CMRP 1330	MT576671	MT584309	MT584328	MT584342	MT584340
*D.ganjae*	CBS 180.91^T^	KC343112	KC343838	KC344080	KC343354	KC343596
*D.ganjae*	PSCG489	MK626955	MK654897	MK691287	MK691202	MK726204
*D.goulteri*	BRIP 55657a^T^	KJ197290	KJ197252	KJ197270	N/A	N/A
*D.gulyae*	BRIP 54025^T^	JF431299	JN645803	N/A	N/A	N/A
*D.gulyae*	BRIP 53158	JF431284	JN645799	N/A	N/A	N/A
*D.guttulata*	CGMCC3.20100^T^	MT385950	MT424685	MT424705	MW022470	MW022491
*D.guttulata*	GZCC 19-0371	MT797178	MT793021	MT793032	MW022471	MW022492
*D.helianthi*	CBS 592.81^T^	KC343115	KC343841	KC344083	KC343357	KC343599
*D.helianthi*	CBS 344.94	KC343114	KC343840	KC344082	KC343356	KC343598
*D.hordei*	CBS 481.92^T^	KC343120	KC343846	KC344088	KC343362	KC343604
*D.infecunda*	CBS 133812^T^	KC343126	KC343852	KC344094	KC343368	KC343610
*D.infertilis*	CBS 230.52^T^	KC343052	KC343778	KC344020	KC343294	KC343536
*D.infertilis*	CPC 20322	KC343053	KC343779	KC344021	KC343295	KC343537
*D.juglandigena*	GUCC 422.16^T^	OP581229	OP688534	OP688559	N/A	N/A
*D.juglandigena*	GUCC 422.161	OP581230	OP688535	OP688560	N/A	N/A
*D.kyushuensis*	STE-U2675^T^	AF230749	N/A	N/A	N/A	N/A
*D.kyushuensis*	ch-D-1	AB302250	N/A	N/A	N/A	N/A
*D.leucospermi*	CBS 111980^T^	N/A	KY435632	KY435673	KY435663	KY435653
*D.leucospermi*	CAA763	MK792291	MK828064	MK837915	MK883823	MK871433
*D.longicolla*	FAU599^T^	KJ590728	KJ590767	KJ610883	KJ612124	KJ659188
*D.longicolla*	CBS 100.87	KC343196	KC343922	KC344164	KC343438	KC343680
*D.longispora*	CBS 194.36^T^	KC343135	KC343861	KC344103	KC343377	KC343619
*D.lusitanicae*	CBS 123213^T^	KC343137	KC343863	KC344105	KC343379	KC343621
*D.lusitanicae*	CBS 123212	KC343136	KC343862	KC344104	KC343378	KC343620
*D.machilii*	SAUCC194.111^T^	MT822639	MT855951	MT855836	MT855718	MT855606
*D.mayteni*	CBS 133185^T^	KC343139	KC343865	KC344107	KC343381	KC343623
*D.megalospora*	CBS 143.27^T^	KC343140	KC343866	KC344108	KC343382	KC343624
*D.melonis*	CBS 507.78^T^	KC343142	KC343868	KC344110	KC343384	KC343626
*D.melonis*	FAU640	KJ590702	KJ590741	KJ610858	KJ612099	KJ659184
*D.melonis*	ZHKUCC 20-0014	MT355684	MT409338	MT409292	MT409314	N/A
***D.melonis***	**MFLUCC 24-0522**	**OR936658**	**PQ774278**	**PQ774285**	**N/A**	**PQ774293**
***D.melonis***	**MFLUCC 23–0300**	**PQ777476**	**PQ774277**	**PQ774284**	**PQ774290**	**N/A**
*D.middletonii*	BRIP 54884e^T^	KJ197286	KJ197248	KJ197266	N/A	N/A
*D.middletonii*	BRIP 57329	KJ197285	KJ197247	KJ197265	N/A	N/A
*D.minusculata*	CGMCC3.20098^T^	MT385957	MT424692	MT424712	MW022475	MW022499
*D.minusculata*	GZCC 19-0345	MT797184	MT793027	MT793038	MW022476	MW022500
*D.monetii*	MF Ha18-048^T^	MW008493	MW008515	MW008504	MZ671938	MZ671964
*D.monetii*	MF Ha18-049	MW008494	MW008516	MW008505	MZ671939	MZ671965
*D.morindendophytica*	ZHKUCC 22-0069^T^	ON322897	ON315053	ON315087	N/A	ON315027
*D.morindendophytica*	ZHKUCC 22-0070	ON322898	ON315054	ON315088	N/A	ON315028
*D.myracrodruonis*	URM 7972^T^	MK205289	MK213408	MK205291	MK205290	N/A
*D.neoarctii*	CBS 109490^T^	KC343145	KC343871	KC344113	KC343387	KC343629
*D.novem*	CBS 127270^T^	KC343156	KC343882	KC344124	KC343398	KC343640
*D.novem*	CBS 127271	KC343157	KC343883	KC344125	KC343399	KC343641
*D.novem*	PL42	JQ697843	JQ697856	N/A	N/A	N/A
*D.orixae*	KUNCC 21-10714^T^	OK283041	N/A	N/A	OK484485	OK484486
*D.orixae*	GZCC 21-1085	OL889852	OL944724	OL944726	N/A	N/A
*D.ovalispora*	CGMCC3.17256^T^	KJ490628	KJ490507	KJ490449	N/A	KJ490570
*D.oxe*	CBS 133186^T^	KC343164	KC343890	KC344132	KC343406	KC343648
*D.oxe*	CBS 133187	KC343165	KC343891	KC344133	KC343407	KC343649
*D.pachirae*	CDA 728^T^	MG559537	MG559539	MG559541	MG559535	N/A
*D.pachirae*	CDA 730	MG559538	MG559540	MG559542	MG559536	N/A
*D.paranensis*	CBS 133184^T^	KC343171	KC343897	KC344139	KC343413	KC343655
*D.paranensis*	LMICRO417	KY461115	KY461116	N/A	N/A	N/A
*D.passiflorae*	CBS 132527^T^	JX069860	N/A	N/A	N/A	KY435654
*D.passiflorae*	CAA953	MN190308	MT309430	MT309456	MT309447	MT309439
*D.pedratalhadensis*	URM 8304^T^	PP192073	PP430438	PP402232	N/A	PP421129
*D.pedratalhadensis*	FCCUFG 49	PP192075	N/A	N/A	PP408217	PP421131
*D.phaseolorum*	AR4203^T^	KJ590738	KJ590739	KJ610893	KJ612135	KJ659220
*D.pseudobauhiniae*	MFLUCC 17-1669^T^	MF190119	MF377598	N/A	N/A	N/A
*D.pseudobauhiniae*	MFLUCC 17-1670	MF190118	MF377599	N/A	N/A	N/A
*D.quercicola*	CSUFTCC104^T^	ON076567	ON081659	N/A	ON081670	ON081667
*D.quercicola*	CSUFTCC105	ON076568	ON081660	N/A	ON081671	ON081668
*D.racemosae*	CBS 143770^T^	MG600223	MG600225	MG600227	MG600219	MG600221
*D.raonikayaporum*	CBS 133182^T^	KC343188	KC343914	KC344156	KC343430	KC343672
*D.raonikayaporum*	MFLUCC 14-1133	KU712448	KU749368	KU743987	KU749355	N/A
*D.raonikayaporum*	MFLUCC 14-1136	KU712449	KU749369	KU743988	KU749356	N/A
*D.rosae*	MFLUCC 17-2658^T^	MG828894	N/A	MG843878	MG829273	N/A
*D.rosae*	MFLUCC 17-2574	MG906793	MG968954	MG968952	N/A	N/A
*D.rosiphthora*	COAD 2914^T^	N/A	QOI91674	N/A	QOI91672	N/A
*D.sackstonii*	BRIP 54669b^T^	KJ197287	KJ197249	KJ197267	N/A	N/A
*D.schini*	CBS 133181^T^	KC343191	KC343917	KC344159	KC343433	KC343675
*D.schini*	LGMF 910	KC343192	KC343918	KC344160	KC343434	KC343676
*D.schoeni*	MFLU 15-1279^T^	KY964226	KY964182	KY964109	KY964139	N/A
*D.schoeni*	MFLU 15-2609	KY964229	KY964185	KY964112	KY964141	N/A
*D.sclerotioides*	CBS 296.67^T^	MH858974	KC343919	KC344161	KC343435	KC343677
*D.sclerotioides*	CBS 710.76	KC343194	KC343920	KC344162	KC343436	KC343678
*D.serafiniae*	BRIP 55665a^T^	KJ197274	KJ197236	KJ197254	N/A	N/A
*D.serafiniae*	BRIP 54136	KJ197273	KJ197235	KJ197253	N/A	N/A
*D.siamensis*	MFLUCC 10-0573a^T^	JQ619879	JX275393	JX275429	JX197423	N/A
*D.siamensis*	MFLUCC 12-0300	KT459417	KT459451	KT459435	KT459467	N/A
*D.sojae*	FAU635^T^	KJ590719	KJ590762	KJ610875	KJ612116	KJ659208
*D.sojae*	CBS 116019	KC343175	KC343901	KC344143	KC343417	KC343659
*D.stewartii*	CBS 193.36	MH867279	GQ250324	JX275421	JX197415	N/A
*D.stewartii*	MN1	KX668416	KX852355	N/A	N/A	N/A
*D.submersa*	CGMCC3.24297^T^	OP056717	OP150556	OP150633	OP150710	OP150786
*D.submersa*	GZCC 22-0007	OP056718	OP150557	OP150634	OP150711	OP150787
*D.subordinaria*	CBS 464.90^T^	KC343214	KC343940	KC344182	KC343456	KC343698
*D.subordinaria*	CBS 101711	KC343213	KC343939	KC344181	KC343455	KC343697
*D.tarchonanthi*	CBS 146073^T^	MT223794	N/A	MT223733	N/A	MT223759
*D.tecomae*	CBS 100547^T^	KC343215	KC343941	KC344183	KC343457	KC343699
*D.tectoendophytica*	MFLUCC 13-0471^T^	KU712439	KU749367	KU743986	KU749354	N/A
*D.tectonendophytica*	LC8115	KY491550	KY491560	KY491570	N/A	N/A
*D.terebinthifolii*	CBS 133180^T^	KC343216	KC343942	KC344184	KC343458	KC343700
*D.terebinthifolii*	LGMF907	KC343217	KC343943	KC344185	KC343459	KC343701
***D.thailandica***	**MFLUCC 24-0523** ^T^	**OR946374**	**PQ774276**	**PQ774283**	**N/A**	**PQ774292**
***D.thailandica***	**MFLUCC 23–0299**	**PQ777475**	**PQ774275**	**PQ774282**	**PQ774289**	**N/A**
*D.thunbergiicola*	MFLUCC 12-0033^T^	KP715097	KP715098	N/A	N/A	N/A
*D.tulliensis*	BRIP 62248a^T^	KR936130	KR936133	KR936132	N/A	N/A
*D.tulliensis*	JZB320128	MK335814	MK523573	MK500152	MK500240	N/A
*D.tulliensis*	MFLUCC 14-1139	KU712438	KU749366	KU743985	KU749353	N/A
***D.tulliensis***	**MFLUCC 24-0524**	**PQ777478**	**PQ774280**	**PQ774287**	**N/A**	**PQ774294**
***D.tulliensis***	**MFLUCC 23–0301**	**PQ777477**	**PQ774279**	**PQ774286**	**PQ774291**	**N/A**
*D.ueckerae*	FAU656^T^	KJ590726	KJ590747	KJ610881	KJ612122	KJ659215
*D.ueckerae*	BRIP 54736j	KJ197282	KJ197244	KJ197262	N/A	N/A
*D.unshiuensis*	ZJUD50^T^	KJ490585	KJ490464	KJ490406	N/A	KJ490527
*D.unshiuensis*	PSCG339	MK626928	MK654879	MK691300	MK691181	MK726188
*D.vangoghii*	MF Ha18-045^T^	MW008491	MW008513	MW008502	MZ671936	MZ671962
*D.vangoghii*	MF Ha18-046	MW008492	MW008514	MW008503	MZ671937	MZ671963
*D.vargemgrandensis*	URM 8784^T^	PP192069	PP430456	PP421092	PP421068	PP421135
*D.vexans*	CBS 127.14	KC343229	KC343955	KC344197	KC343471	KC343713
*D.vexans*	FAU597	KJ590734	KJ590774	KJ610889	KJ612131	KJ659216
*D.vochysiae*	LGMF1583^T^	MG976391	MK007526	MK007527	MK007528	MK033323
*D.yunnanensis*	CGMCC3.18289^T^	KX986796	KX999188	KX999228	KX999290	KX999267
*D.yunnanensis*	LC8107	KY491542	KY491552	KY491562	KY491572	N/A

The newly generated sequences are in bold. Type and reference collections are denoted with ^“T,”^ while missing data are shown as “N/A”. * The species name is invalid but included for taxon sampling.

**Table 2.** GenBank accession numbers of the taxa used in the phylogenetic analyses of Xylariales.

Species	Strain	ITS	LSU	*rpb2*	*tub2*
*Albicollumvincensii*	CBS 147286^T^	ON869297	ON869297	ON808475	ON808519
*Amphirosellinianigrospora*	HAST 91092308^T^	GU322457	N/A	GQ848340	GQ495951
*Anthostomellahelicofissa*	MFLUCC 14-0173^T^	MW240653	MW240583	KP340534	KP406617
*Anthostomellalamiacearum*	MFLU18-0101^T^	MW240669	MW240599	MW658648	N/A
*Anthostomelloidesbrabeji*	CBS 110128	EU552098	EU552098	N/A	N/A
*Anthostomelloideskrabiensis*	MFLUCC 15-0678^T^	KX305927	KX305928	KX305929	N/A
*Anthostomelloidesleucospermi*	CBS 110126^T^	EU552100	EU552100	N/A	N/A
*Barrmaeliamacrospora*	CBS 142768^T^	KC774566	KC774566	MF488995	MF489014
*Biscogniauxianummularia*	MUCL 51395^T^	KY610382	KY610427	KY624236	KX271241
*Chaetomiumelatum*	CBS 374.66	KC109758	KC109758	KF001820	KC109776
*Circinotrichumcircinatum*	CBS 148326	ON400743	ON400796	ON399328	N/A
*Circinotrichummaculiforme*	CBS 140016^T^	KR611874	KR611895	ON399338	N/A
*Clypeosphaeriamamillana*	CBS 140735^T^	KT949897	KT949897	MF489001	MH704637
*Clypeosphaeriamamillana*	WU 33599	KT949898	KT949898	N/A	N/A
*Clypeosphaeriaoleae*	CPC 36779	MN562130	MN567637	N/A	N/A
*Coniocessiamaxima*	CBS 593.74^T^	GU553332	MH878275	N/A	N/A
*Coniocessianodulisporioides*	CBS 281.77^T^	MH861061	MH872831	N/A	N/A
*Dematophorabunodes*	CBS 124028	MN984619	MN984625	N/A	MN987245
*Didymobotryumrigidum*	JCM 8837^T^	LC228650	LC228707	N/A	N/A
*Digitodochiumamoenum*	CBS 147285^T^	ON869303	ON869303	ON808481	ON808525
*Digitodochiumrhodoleucum*	NBRC 32296	LC146732	LC146732	N/A	N/A
*Emarceacastanopsidicola*	CBS 117105	AY603496	MK762717	MK791285	MK776962
*Emarceaeucalyptigena*	CBS 139908	KR476733	MK762718	MK791286	MK776963
*Entalbostromaerumpens*	ICMP 21152^T^	KX258206	N/A	KX258204	KX258205
*Entoleucamammata*	JDR 100	GU300072	N/A	GQ844782	GQ470230
*Entosordariaperfidiosa*	CBS 142773^T^	MF488993	MF488993	MF489003	MF489021
*Fasciatisporaarengae*	MFLUCC 15-0326a^T^	MK120275	MK120300	MK890794	MK890793
*Fasciatisporacocoes*	MFLUCC 18-1445^T^	MN482680	MN482675	MN481517	MN505154
*Graphostromaplatystomum*	CBS 270.87^T^	JX658535	DQ836906	KY624296	HG934108
*Gyrothrixverticillata*	CBS 148806	ON400759	ON400813	ON399318	N/A
*Haloroselliniakrabiensis*	MFLU 17-2596^T^	MN047119	MN017883	N/A	MN431493
*Hansfordiapruni*	CBS 194.56^T^	MK442585	MH869122	KU684307	N/A
*Hansfordiapulvinata*	CBS 144422	MK442587	MK442527	N/A	N/A
*Helicogermslitaclypeata*	MFLU 18-0852^T^	MW240666	MW240596	MW658647	MW775614
*Hypocoprarostrata*	NRRL 66178	KM067909	KM067909	N/A	N/A
*Hypocoprazeae*	MFLU 18-0809^T^	MW240671	MW240601	MW658650	MW775616
*Hypocreodendronsanguineum*	J.D.R.169^T^	GU322433	N/A	GQ844819	GQ487710
*Hypomontagnellamonticulosa*	MFLUCC 18-0362	MN337231	MN336235.2	MN366246	MN509783
*Muscodorthailandicus*	MFLUCC 17-2669	MK762707	MK762714	MK791283	MK776960
*Muscodorziziphi*	MFLUCC 17-2662	MK762705	MK762712	MK791281	MK776958
*Jackrogersellamultiformis*	CBS 119016^T^	KC477234	KY610473	KY624290	KX271262
*Kretzschmariadeusta*	CBS 163.93	KC477237	KY610458	KY624227	KX271251
*Kretzschmariellaculmorum*	JDR 88	KX430043	N/A	KX430045	KX430046
*Linosporopsisischnotheca*	CBS 145761^T^	MN818952	MN818952	MN820708	MN820715
*Magnostiolatamucida*	MFLU 19-2133^T^	MW240673	MW240603	MW658652	MW775618
*Melanographiumphoenicis*	MFLUCC 18-1481^T^	MN482677	MN482678	N/A	N/A
*Melanographiumsmilacis*	MFLU 21-0075^T^	MZ538514	MZ538548	N/A	N/A
*Nemaniaserpens*	HAST 235	GU292820	N/A	GQ844773	GQ470223
*Neoanthostomellafici*	MFLU 19-2765^T^	MW114390	MW114445	MW177711	N/A
*Neoxylariajuruensis*	HAST 92042501	GU322439	N/A	GQ844825	GQ495932
*Nigropunctatabambusicola*	MFLU 19-2145^T^	MW240664	MW240594	MW658646	N/A
*Nigropunctatanigrocircularis*	MFLU 19-2130^T^	MW240661	MW240591	N/A	MW775612
*Occultithecaananasi*	MFLU 23-0251^T^	OR438426	OR438886	N/A	N/A
*Occultithecaananasi*	MFLUCC 23-0120	OR438427	OR438887	OR634962	OR538094
***Occultithecachiangraiensis***	**MFLU 24-0414** ^T^	**PQ777479**	**PQ778042**	**PQ774295**	**PQ774288**
*Occultithecarosae*	HKAS 102393^T^	MW240672	MW240602	MW658651	MW775617
*Podosordariamexicana*	WSP 176	GU324762	N/A	GQ853039	GQ844840
*Poroniapunctata*	CBS 656.78^T^	KT281904	KY610496	KY624278	KX271281
*Pseudoanthostomellapini-nigrae*	MFLUCC 16-0478^T^	KX533453	KX533454	KX789492	N/A
*Pseudoceratocladiumpolysetosum*	FMR 10750^T^	KY853430	KY853490	ON399348	N/A
*Roselliniachiangmaiensis*	MFLUCC 15-0015^T^	KU246226	KU246227	N/A	N/A
*Rosellinialamprostoma*	HAST 89112602	EF026118	N/A	GQ844778	EF025604
*Sarcoxyloncompunctum*	CBS 359.61	KT281903	KT281898	KY624230	KX271255
*Sordariafimicola*	CBS 723.96	MH862606	MH874231	DQ368647	N/A
*Spiririmagaudefroyi*	CBS 147284^T^	ON869320	ON869320	ON808497	ON808541
*Spirodecosporamelnikii*	MAFF 247746^T^	LC731937	LC731946	LC731955	N/A
*Spirodecosporapaulospiralis*	MAFF 247749^T^	LC731940	LC731949	LC731957	N/A
*Stromatoneurosporaphoenix*	BCC 82040	MT703666	MT735133	MT742605	MT700438
*Vamsapriyaindica*	MFLUCC 12-0544	KM462839	KM462840	KM462841	KM462838
*Xenoanthostomellachromolaenae*	MFLUCC 17-1484^T^	MN638863	MN638848	MN648729	N/A
*Xenoanthostomellacycadis*	CBS 137969^T^	KJ869121	KJ869178	ON399350	N/A
*Xylariaacuminatilongissima*	HAST 95060506^T^	EU178738	N/A	GQ853028	GQ502711
*Xylariaarbuscula*	CBS 126415	KY610394	KY610463	KY624287	KX271257
*Xylariabotuliformis*	HAST 89091627	MN089652	N/A	MN095399	MN095400
*Xylariabrunneovinosa*	HAST 720^T^	EU179862	N/A	GQ853023	GQ502706
*Xylariaellisii*	DAOM 628556^T^	MN218820	MN218817	MN216186	N/A
*Xylariaeucalypti*	CPC 36723	MN562127	MN567634	N/A	N/A
*Xylariahypoxylon*	CBS 122620^T^	AM993141	KM186301	KM186302	KM186300

The newly generated sequences are in bold. Type and reference collections are denoted with ^“T,”^ while missing data are shown as “N/A”.

### ﻿Alignments and phylogenetic analyses

Consensus sequences were assembled using Geneious Prime 2025 (Biomatters Ltd., Auckland, New Zealand) and were used for BLASTn search against the NCBI nucleotide non-redundant database (Sayers et al. 2022). For *Diaporthe*, sequences were downloaded from GenBank (Table 1) following the classification in Dissanayake et al. (2024). For Xylariales, related sequences were downloaded from GenBank (Table 2) based on recent publications (Samarakoon et al. 2022; Sugita et al. 2022; Voglmayr et al. 2022). The sequences were aligned using MAFFT version 7 (Katoh et al. 2019) with minimal adjustment of any ambiguous nucleotides using AliView version 1.26 (Larsson 2014). The alignments were concatenated using SequenceMatrix version 1.8 (Vaidya et al. 2011).

Maximum likelihood analyses (ML), including 1000 bootstrap pseudoreplicates, were performed at the CIPRES web portal (Miller et al. 2017) using RAxML version 8.2.12 (Stamatakis 2014). The general time reversible (GTR) model with a discrete gamma distribution plus invariant site (GTR + I + G) was used as the nucleotide substitution model. The best model for each gene was determined in JModelTest version 2.1.10 (Darriba et al. 2012) for the Bayesian analysis. The Bayesian inference posterior probabilities (BPP) distribution (Zhaxybayeva and Gogarten 2002) was estimated by Markov Chain Monte Carlo sampling (MCMC) in MrBayes version 3.2.2 on XSEDE (Ronquist et al. 2012) with four runs of MCMC for 1,000,000 generations, sampling trees every 100^th^ generation. The first 25% of trees were excluded as burn-in, and the remaining trees were used to calculate posterior probabilities (BPP). The trees were visualised using FigTree version 1.4.4 (Rambaut 2012) and edited using Adobe Illustrator® CS6 (Adobe Systems, USA).

### ﻿Genealogical concordance phylogenetic species recognition analysis

The closely related strains were further analysed using the genetic distances by performing a pairwise homoplasy index test (Φw) (Taylor et al. 2000; Bruen et al. 2006). A pairwise homoplasy index (PHI) test was performed in SplitsTree (CE 6.0.0) using Kimura’s two-parameter (K2P) models for low genetic distance datasets (Huson and Bryant 2024). LogDet transformation was applied for the average of nucleotide frequencies and splits decomposition graph options (Gu and Li 1996a, b; Taylor et al. 2000; Bruen et al. 2006; Huson and Bryant 2006; Gioan and Paul 2012; Nishimaki and Sato 2019). The standard deviation of split frequencies PHI test result (Φw) < 0.05 indicates significant recombination within the dataset.

## ﻿Results

### ﻿Phylogenetic analyses of section sojae

**Figure 1.**
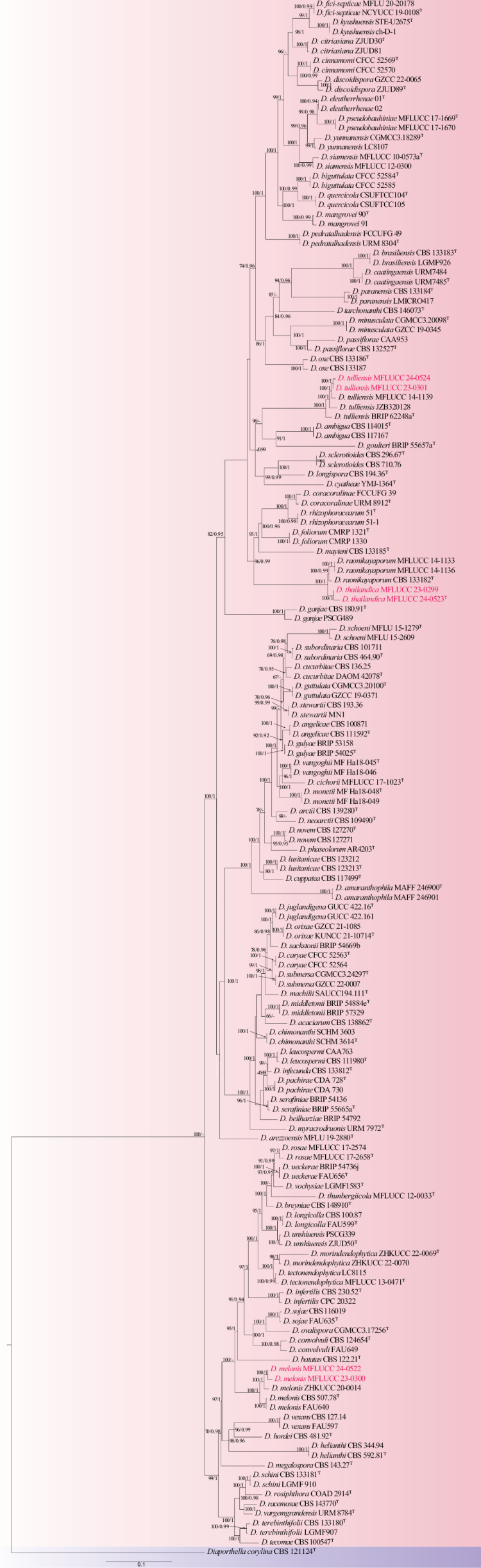
Phylogram of the *Diaporthesojae* species complex generated from a maximum likelihood analysis based on the combined ITS, *tef1*, *tub2*, *cal*, and *his3* sequence data. *Diaporthellacorylina* (CBS 121124) was used as the outgroup. Bootstrap support values ≥ 60% ML/≥0.90 BPP are given at the nodes. The newly generated taxa are indicated in red. The holotype/ex-type strains are denoted with ^T^.

The phylogeny represents taxa from section sojae based on the concatenated dataset of ITS, *tef1*, *tub2*, *cal*, and *his3* sequences. The combined sequence alignment comprised 159 taxa with 3279 characters, including gaps (ITS: 1–587, *tef1*: 588–1131, *tub2*: 1132–2067, *cal*: 2068–2682, *his3*: 2683–3279). The ML and BI analyses showed similar topologies (Fig. 1). The best scoring ML tree had a final likelihood value of -47785.673. The matrix had 1463 constant sites, 1408 parsimony informative sites, and 2202 distinct site patterns. Estimated base frequencies were as follows: A = 0.213, C = 0.325, G = 0.239, T = 0.224, substitution rates: AC = 1.16629, AG = 3.42597, AT = 1.16629, CG = 1.00000, CT = 4.44139, GT = 1.000, gamma distribution shape parameter = 0.954, and tree length = 7.506.

#### ﻿Sordariomycetes O.E. Erikss. & Winka

**Diaporthales Nannf.**

**Diaporthaceae Höhn. ex Wehm.**

##### ﻿ Diaporthe melonis

Taxon classificationFungiDiaporthalesDiaporthaceae

Beraha & M.J. O’Brien, Phytopath. Z. 94(3): 205 (1979)

2F84ED18-BD34-5418-9713-D10835287DDA

Index Fungorum: IF312933

Facesoffungi Number: FoF17285

Fig. 2

###### Description.

***Saprobic*** on dead unidentified branch. ***Sexual morph***: not observed. ***Asexual morph*: *Conidiomata*** 148–374 × 128–338 µm high (x̄ = 250 × 225 µm, n = 15), pycnidial, mostly scattered, immersed, slightly erumpent through the host surface, discoid or subglobose, with a solitary undivided locule. ***Conidiophores*** reduced to conidiogenous cells. ***Alpha conidiogenous cells*** 5.7–25 × 1.1–2.5 µm (x̄ = 15.6 × 1.7 µm, n = 50), hyaline, rarely branched, mostly aseptate, densely aggregated, cylindrical, straight to slightly curved and smooth. ***Alpha conidia*** 5–7.3 × 1.9–2.7 µm (x̄ = 6.3 × 2.3 µm, n = 40), unicellular, fusiform to ellipsoidal, apex and base rounded, hyaline, smooth, bi-guttulate. ***Beta conidiogenous cells*** 6.2–16 × 1.6–2.6 µm (x̄ = 9.4 × 2.1 µm, n = 40), phialidic, subcylindrical, tapering towards the apex, hyaline. ***Beta conidia*** 19–27 × 1–2 µm (x̄ = 23 × 1.5 µm, n = 40), filiform, aseptate, hyaline, smooth-walled, straight from base, and curve at apex. ***Gamma conidia*** not observed.

###### Culture characteristics.

Colonies on PDA, reaching 20 mm diam., after 3 weeks at 25 °C, initially white, turning beige after 7–10 days, flat, felty with a thick texture at the centre and marginal area, lacking aerial mycelium; reverse, glossy grey, radiating outwardly.

###### Material examined.

Thailand, Chiang Rai Province, Muang District, on a dead unidentified dicot branch, 16 January 2023, J. Louangphan, CR1-02 (MFLU 23–0474); living culture MFLUCC 24–0522 = MFLUCC 23–0300.

###### Hosts.

*Annonasquamosa* (Annonaceae), *Berberisaristata* (Berberidaceae), *Carapaguianensis* (Meliaceae), *Citrusgrandis* cv. Tomentosa (Rutaceae), *Cucumismelo* (Cucurbitaceae), *Glottidium* sp. (Fabaceae), *Glycinemax*, *G.soja* (Fabaceae), unidentified branch (Dong et al. 2021a, b; Hongsanan et al. 2023; This study).

**Figure 2.**
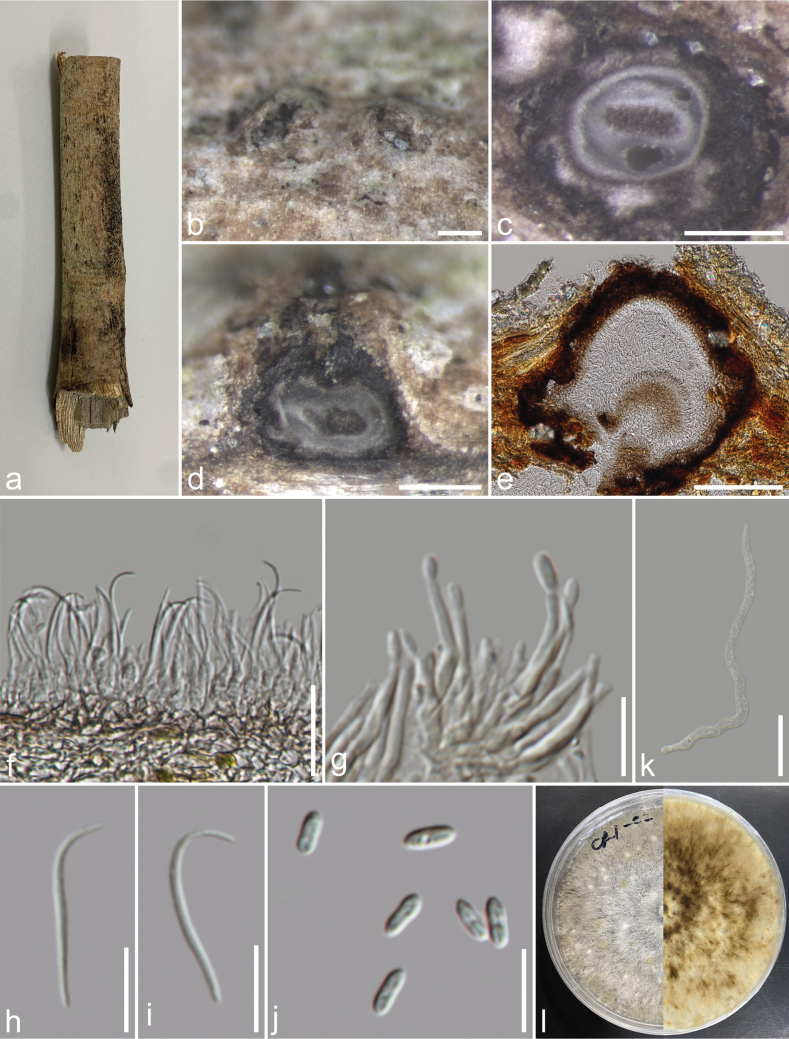
*Diaporthemelonis* (MFLU 23–0474) **a** host substrate **b** conidiomata on substrate **c** transverse section of conidioma **d, e** vertical section through conidiomata **f, g** conidiophores and conidiogenous cells **h, i** beta conidia **j** alpha conidia **k** a germinated conidium **l** front and reverse view of the colony on PDA. Scale bars: 200 μm (**b–d**); 100 μm (**e**); 20 μm (**f, k**); 10 μm (**g, h–j**).

###### Distribution.

China, Myanmar, India, Indonesia, Japan, Thailand, the United States (Dong et al. 2021a, b; Hongsanan et al. 2023; this study).

###### Notes.

Our isolates (MFLUCC 23–0300 and MFLUCC 24–0522) clustered with *D.melonis* isolates (CBS 507.78, FAU640, and ZHKUCC 20-0014) with 100% ML/1.00 BPP support (Fig. 1). Our isolate has a similar morphology to *D.melonis* but differs in having smaller conidiomata (148–374 µm vs. 100–500 µm diam.) and smaller alpha conidia (6.3 × 2.3 µm vs. 8.3 × 2.6 µm) (Beraha and O’Brien 1979). Our isolate has a beige culture compared to the brown culture of *D.melonis* (Beraha and O’Brien 1979). Our isolate also differs from *D.melonis* (*D.guangdongensis* ZHKUCC 20-0014) in the size of conidiomata (128–338 × 148–374 µm *vs.* 130–515 × 100–390 µm), alpha conidia (5–7.3 × 1.9–2.7 µm vs. 6–8 × 2–4 µm), and beta conidia (19–27 × 1–2 µm vs. 14–35 × 1–2 µm) (Dong et al. 2021a, b). Therefore, we report our isolate as a new geographical record of *D.melonis* from Thailand.

##### ﻿ Diaporthe thailandica

Taxon classificationFungiDiaporthalesDiaporthaceae

Louangphan, Phukhams., K.D. Hyde & Bhunjun sp. nov.

15198841-8AEB-533A-A9CC-4F5E663F7153

Index Fungorum: IF903043

Facesoffungi Number: FoF17286

Figs 3 , 4

###### Etymology.

The name refers to the country where the holotype was collected.

###### Holotype.

MFLU 23–0473.

###### Description.

***Saprobic*** on decaying dicot, visible as black necks immerging through the host surface. ***Sexual morph*: *Ascomata*** 328–495 × 303–371 µm (x̄ = 400 × 343 µm, n = 10), immersed in the host epidermis, globose to sub-globose, solitary or occur in clusters, black, ostiolate, papillate. ***Ostiole neck*** 220 × 86 µm, long, filled with periphysate. ***Peridium*** 20–50 µm wide, composed of several layers of cells of *textura angularis*, outer layers dark brown and inner layers hyaline to brown, thin-walled. ***Paraphyses*** 3.2–6.6 µm (n = 20), thin-walled, 2–4-septate, hyaline, wide at base, tapering towards the apex. ***Asci*** 45–58.9 × 8.6–12.7 µm (x̄ = 51.5 × 10.5 µm, n = 40), unitunicate, 8-spored, clavate to subclavate, straight to slightly curved, sessile, with a J-, apical ring. ***Ascospores*** 11–15.5 × 3.9–5.6 µm (x̄ = 13.5 × 4.7 µm, n = 40) L/W = 2.8, overlapping uniseriate to biseriate, 1-septate, constricted at the septum, ellipsoidal, smooth-walled, 2–4-guttulate, straight, hyaline, without appendages or a mucilaginous sheath. ***Asexual morph*** on PDA: ***Conidiomata*** 500–700 × 300–600 µm (x̄ = 580 × 480 µm, n = 10), pycnidial, scattered or aggregated, globose or variable in shape, ostiolate with prominent neck, dark brown to black, pycnidal wall brown, consisting of thick-walled cells of *textura angularis*, conidial mass globose, initially hyaline to yellowish, becoming white to cream, conidial droplets exuding from central ostioles. ***Conidiophores*** 10–29.5 × 1.3–2.5 μm (x̄ = 16.9 × 1.9 µm, n = 40), ampulliform to subcylindrical, filiform, branched to unbranched, 1–3-septate, hyaline, smooth, straight or slightly curved, wider at base, tapering towards the apex. ***Conidiogenous cells*** 2.1–8.1 × 1–2.3 μm (x̄ = 4.2 × 1.5 µm, n = 40), subcylindrical, filiform, straight to curved, tapering towards the apex, collarette not flared, hyaline. ***Alpha conidia*** 5.3–8.8 × 2.3–3.5 μm (x̄ = 7.3 × 2.9 μm, n = 40), ellipsoid, apex bluntly rounded, base obtuse to subtruncate, smooth, hyaline, bi- to multi-guttulate. ***Beta conidia*** 8.5–18.5 × 1.2–2 μm (x̄ = 13.3 × 1.7 μm, n = 40), filiform, flexible to slightly curved, hyaline, base subtruncate, and aseptate. ***Gamma conidia*** not observed.

**Figure 3.**
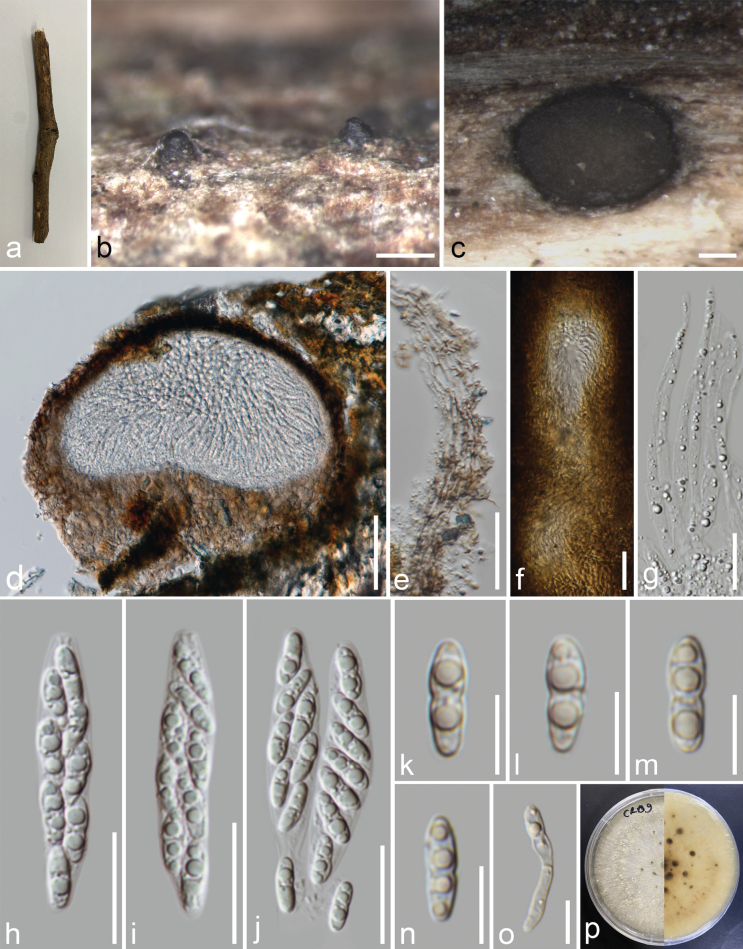
*Diaporthethailandica* (MFLU 23–0473, holotype) **a** host substrate **b, c** ascomata on host substrate **d** vertical section through ascoma **e** peridium **f** ostiole **g** hamathecium **h–j** asci **k–n** ascospores **o** a germinated ascospore **p** front and reverse view of the colony on PDA. Scale bars: 200 μm (**b**); 100 μm (**c, d**); 20 μm (**e–j**); 10 μm (**k–o**).

**Figure 4.**
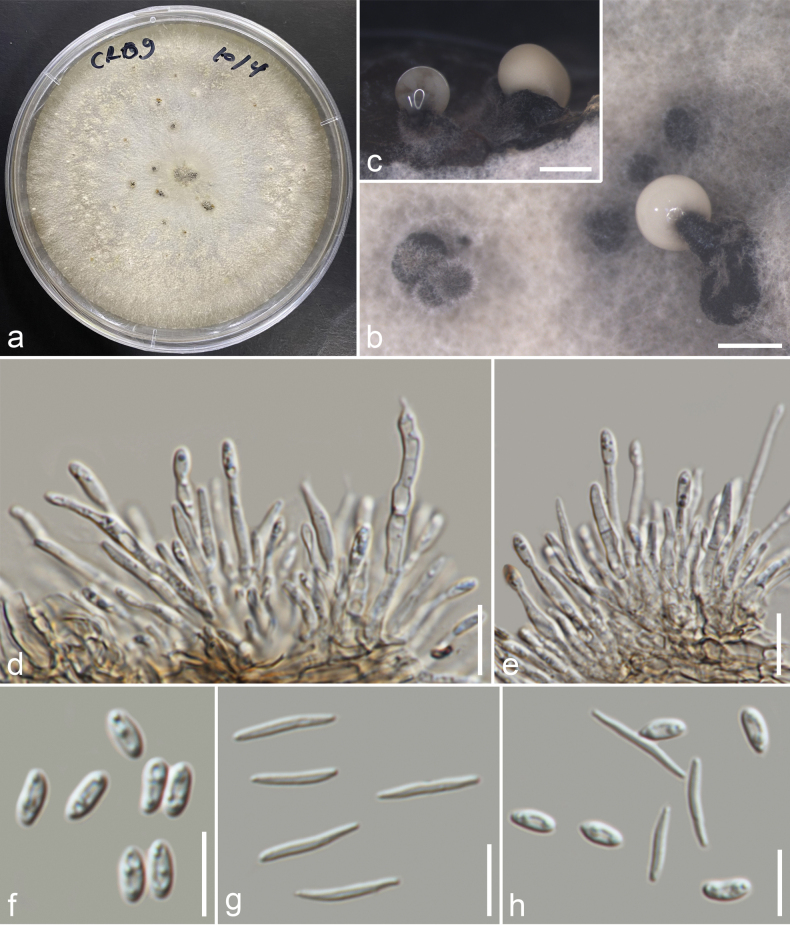
*Diaporthethailandica* (Asexual morph, MFLUCC 24–0523) **a** culture on pda **b, c** conidiomata sporulating on pda **d, e** conidiogenous cells giving rise to conidia **f** alpha conidia **g** beta conidia **h** alpha and beta conidia. Scale bars: 500 µm **(b, c)**; 10 µm **(d–h)**.

###### Culture characteristics.

Colonies on PDA, reaching 40 mm diam., after 2 weeks at 25 °C, initially white, turning pale brown after 7–10 days, radiating to the edge, margin undulate, medium dense, flat or umbonate; reverse, cream, radiating white outwardly with grey patches.

###### Material examined.

Thailand, Chiang Rai Province, Muang District, on a dead unidentified dicot, 16 January 2023, J. Louangphan, CR1-09 (MFLU 23–0473, ***holotype***); ex-type MFLUCC 24–0523 = MFLUCC 23–0299.

###### Host.

Unidentified branch (this study).

###### Distribution.

Thailand (this study).

###### Notes.

**Figure 5.**
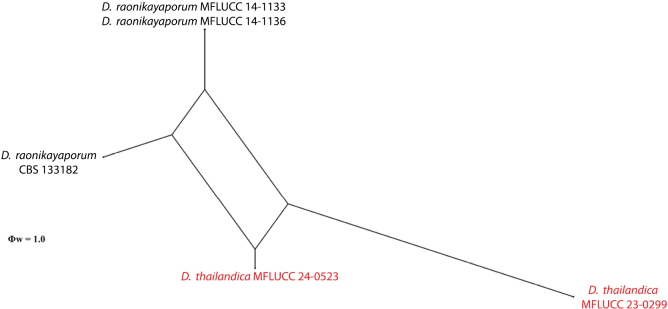
The splits graph from the pairwise homoplasy index (PHI) test generated from the combined ITS, *tef1*, *tub2*, *cal*, and *his3* sequence data of *Diaporthethailandica* (indicated in red) and closely related taxa using both LogDet transformation and splits decomposition. PHI test results (Φw) < 0.05 indicate significant recombination within the dataset.

*Diaporthethailandica* (MFLUCC 23-0299 and MFLUCC 24–0523) formed a sister clade with isolates of *D.raonikayaporum* (CBS 133182, MFLUCC 14–1133, and MFLUCC 14–1136) with 100% ML/1.00 BPP support (Fig. 1). *Diaporthethailandica* differs from *D.raonikayaporum* in its conidiomata (500–700 × 300–600 µm *vs.* 110–200 × 50–130 μm), conidiophores (10–29.5 × 1.3–2.5 μm vs. 16–26 × 2–3 μm), conidiogenous cells (2.1–8.1 × 1–2.3 μm *vs.* 5–10 × 2–3 μm), and beta conidia (8.5–18.5 *vs.* 7–13 μm) (Gomes et al. 2013). *Diaporthethailandica* differs from *D.raonikayaporum* (=*D.neoraonikayaporum*MFLUCC 14–1133) in its conidiomata (500–700 × 300–600 µm vs. 690–1190 × 805–1285 μm), conidiophores (10–29.5 μm *vs.* 15–23 μm), alpha conidia (5.3–8.8 × 2.3–3.5 μm *vs.* 4–6 × 2–3 μm), and beta conidia (8.5–18.5 μm *vs.* 13–21 μm) (Doilom et al. 2017). Gamma conidia was observed in *D.raonikayaporum* (= *D.neoraonikayaporum*) but not in *D.thailandica* (Doilom et al. 2017). *Diaporthethailandica* further differs from *D.raonikayaporum*, which has only been reported as an asexual morph (Gomes et al. 2013; Doilom et al. 2017). Our strain differs significantly (> 2.5%) compared to the sequence data of *D.raonikayaporum* (Table 3). However, our isolate does not have *cal* sequence data, while *his3* sequence data is not available for *D.raonikayaporum* (MFLUCC 14–1133 and MFLUCC 14–1136). A pairwise homoplasy index showed Φw = 1.0 when a genealogical correlation model was applied between *Diaporthethailandica* and *D.raonikayaporum* (Fig. 5). Thus, *Diaporthethailandica* is reported as a new species based on morphology and molecular evidence.

**Table 3.** Pairwise comparison of the sequences of *Diaporthethailandica* and *D.raonikayaporum* isolates (excluding gaps).

Sequences	*D.raonikayaporum* (CBS 133182)	*D.raonikayaporum* (MFLUCC 14-1133)	*D.raonikayaporum* (MFLUCC 14-1136)
ITS	3.4% (18/529)	2.6% (14/529)	3.0% (16/529)
*tef1*	6.5% (21/323)	7.4% (24/323)	8.4% (27/323)
*tub2*	4.3% (18/415)	6.5% (27/415)	7.7% (32/415)
*cal*	−	−	−
*his3*	3.7% (17/450)	−	−

− Data not available.

##### ﻿ Diaporthe tulliensis

Taxon classificationFungiDiaporthalesDiaporthaceae

R.G. Shivas, Vawdrey & Y.P. Tan, Persoonia 35: 301 (2015)

09BDA018-5099-56DA-8350-2C34B63FD1CE

Index Fungorum: IF812896

Facesoffungi Number: FoF16300

Fig. 6

###### Description.

***Saprobic*** on decaying *Bambusa*. ***Sexual morph***: Undetermined. ***Asexual morph*: *Conidiomata*** 91–148 × 311–974 μm (x̄ = 120 × 583 μm, n = 20), pycnidial, scattered or aggregated, embedded in host surface, slightly erumpent through host surface, 1–3-locular conidioma, nearly flat, elongated, discoid, or variable in shape, black, consisting of hyaline, thin-walled cells of *textura angularis*, outer layer thick walled. ***Conidiophores*** reduced to conidiogenous cells. ***Conidiogenous cells*** 4–16 × 1.2–2.6 μm (x̄ = 7.5 × 1.8 μm, n = 40), cylindrical, unbranched, aseptate, smooth, straight or slightly curved, tapering towards the apex, wider at base, hyaline. ***Alpha conidia*** 4.1–7.8 × 1.7–3.1 μm (x̄ = 5.8 × 2.5 μm, n = 40), apex bluntly rounded, 1–2-guttulate, mostly bi-guttulate, oval or oblong to ellipsoid, hyaline, smooth, base obtuse to subtruncate. ***Beta*** and ***Gamma conidia*** not observed.

**Figure 6.**
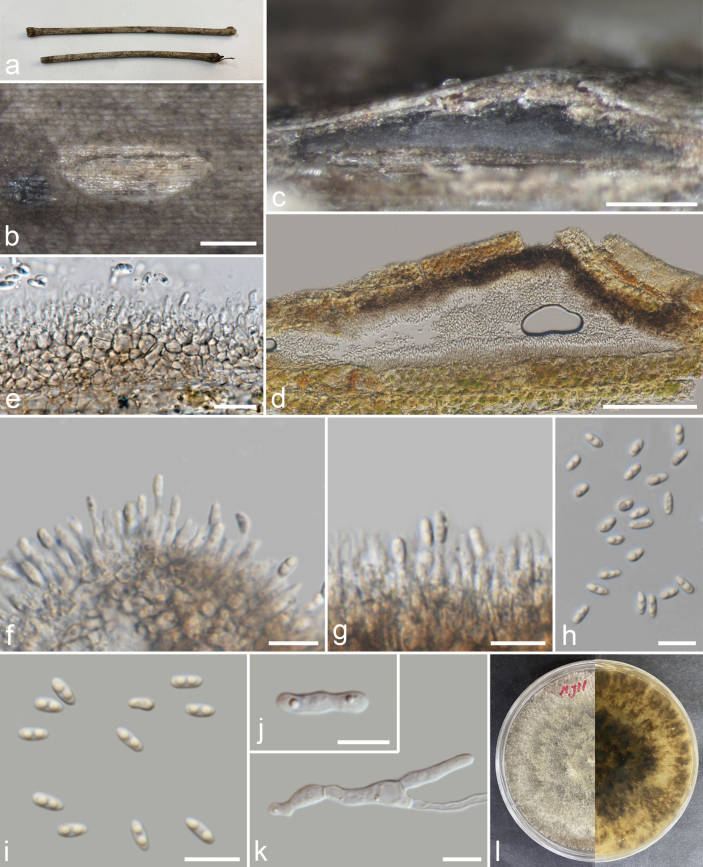
*Diaporthetulliensis* (MFLU 23–0475) **a, b** conidiomata on host **c** longitudinal section through conidioma **d, e** transverse section of conidioma **f, g** conidiogenous cells giving rise to conidia **h, i** alpha conidia **j, k** germinated conidia **l** front and reverse view of the colony on PDA. Scale bars: 200 µm (**b–d**); 20 µm (**e**); 10 µm (**f–k**).

###### Culture characteristics.

Colonies on PDA, reaching 40 mm diam., after 2 weeks at 25 °C, initially white, turning olivaceous grey after 7–10 days, darker at the centre and marginal area, lacking aerial mycelium; reverse, olivaceous grey bordered by dark margins.

###### Material examined.

Thailand, Chiang Mai Province, Mae Taeng District, on dead terrestrial stem of *Bambusa* (Poaceae), 19 November 2022, J. Louangphan, MJ11 (MFLU 23–0475); living culture, MFLUCC 24–0524 = MFLUCC 23–0301.

###### Hosts.

*Actinidia* spp. (Actinidiaceae), *Alangiumkurzii* (Cornaceae), *Bambusa* sp. (Poaceae), *Bougainvilleaglabra* (Nyctaginaceae), *Celtisformosana* (Ulmaceae), *Morindaofficinalis* (Rutaceae), *Tectonagrandis* (Lamiaceae), *Theobromacacao* (Malvaceae), Soil, *Vitisvinifera* (Vitaceae) (Chang et al. 2005; Crous et al. 2015; Bai et al. 2017; Doilom et al. 2017; Yang et al. 2018; Manawasinghe et al. 2019; Tennakoon et al. 2021; Luo et al. 2022; this study).

###### Distribution.

Australia, China, Korea, Thailand (Chang et al. 2005; Crous et al. 2015; Bai et al. 2017; Doilom et al. 2017; Yang et al. 2018; Manawasinghe et al. 2019; Tennakoon et al. 2021; Luo et al. 2022; this study).

###### Notes.

In the phylogenetic analysis, our isolates (MFLUCC 23–0301 and MFLUCC 24–0524) clustered with *D.tulliensis* isolates (MFLUCC 14–1139, JZB320128, and BRIP 62248a) with 100% ML/1.00 BPP support (Fig. 1). Our isolate has a similar morphology to *D.tulliensis* isolates but differs from *D.tulliensis* in the size of conidiomata (up to 500 µm (= *D.celtidis* and *D.tulliensis*) *vs.* up to 510 µm (= *D.hubeiensis*) *vs.* 135–330 μm (= *D.alangii*) *vs.* 50–380 μm (= *D.morindae*) *vs.* 725–820 μm diam. (= *D.tectonae*)) (Crous et al. 2015; Doilom et al. 2017; Yang et al. 2018; Manawasinghe et al. 2019; Luo et al. 2022). Our isolate also differs due to the absence of beta conidia, which has been reported in some *D.tulliensis* isolates (Chang et al. 2005; Crous et al. 2015; Doilom et al. 2017; Manawasinghe et al. 2019). Therefore, we report our isolate as a new host record of *D.tulliensis*.

### ﻿Phylogenetic analyses of Xylariales

**Figure 7.**
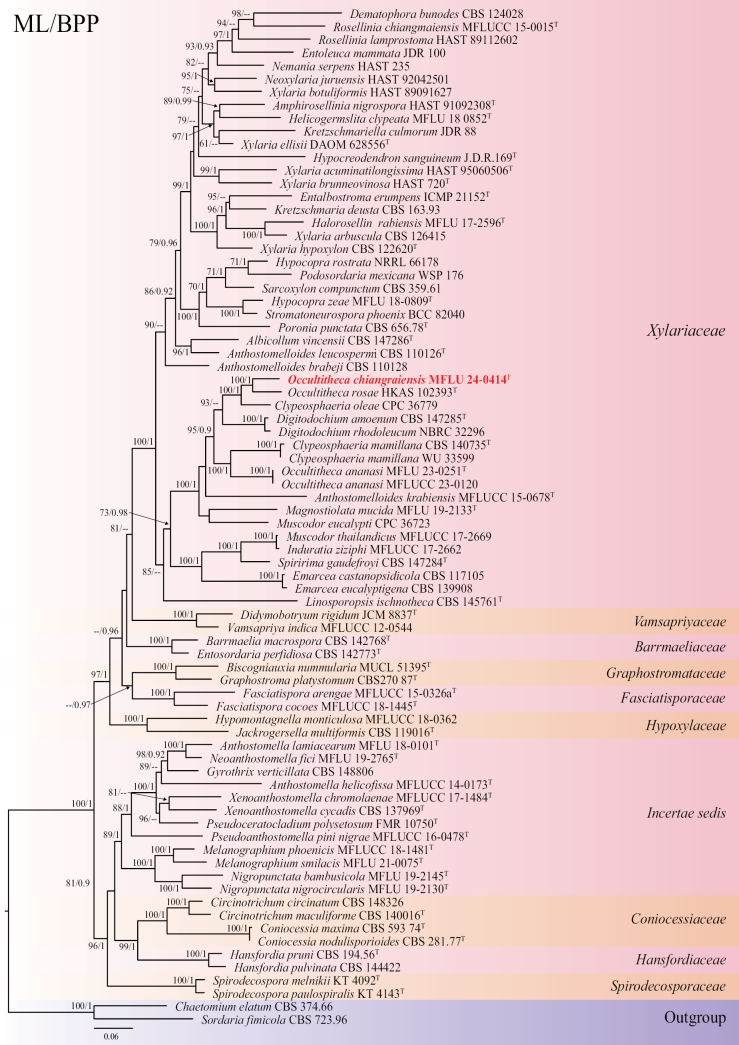
Phylogram generated from maximum likelihood analyses based on combined ITS-LSU-*rpb2*-*tub2* datasets. The tree is rooted with *Chaetomiumelatum* (CBS 374.66) and *Sordariafimicola* (CBS 723.96) as the outgroup taxa. Bootstrap support values ≥ 60% ML/≥0.90 BPP are given at the nodes. The newly generated taxa are indicated in red. The holotype/ex-type strains are denoted with ^T^.

The phylogeny represents selected taxa in Xylariales based on the concatenated dataset of ITS, LSU, *rpb2*, and *tub2* sequences. The combined sequence alignment comprised 78 strains with 3624 characters, including gaps (ITS: 1–578, LSU: 579–1432, *rpb2*: 1433–2573, *tub2*: 2574–3624). The ML and BI analyses of single and multi-gene showed similar topologies. The best scoring ML tree with a final likelihood value of -54607.950 (Fig. 7). The matrix had 1992 constant sites, 1349 parsimony informative sites, and 2026 distinct site patterns. Estimated base frequencies were as follows: A = 0.2405, C = 0.2618, G = 0.2637, T = 0.2337, substitution rates: AC = 1.4137, AG = 3.9195, AT = 1.4543, CG = 1.1208, CT = 7.4615, GT = 1.000, gamma distribution shape parameter = 0.759631, and tree length = 7.506.

### ﻿Xylariales Nannf.

**Xylariaceae Tul. & C. Tul.**

***Occultitheca* J.D. Rogers & Y.M. Ju**

#### ﻿ Occultitheca chiangraiensis

Taxon classificationFungiXylarialesXylariaceae

Louangphan, Phukhams., K.D. Hyde & Bhunjun sp. nov.

AE6F81DB-4828-5A51-9EE2-43BE137F4892

Index Fungorum: IF903235

Facesoffungi Number: FoF17287

Fig. 8

##### Etymology.

The name refers to the province where the holotype was collected.

##### Holotype.

MFLU 24–0414.

##### Description.

***Saprobic*** on early decaying branch. ***Sexual morph*: *Ascomata*** 220–342 × 228–395 μm (x̄ = 290 × 324 μm, n = 15), immersed, solitary, scattered, globose to subglobose, erumpent through host surface, visible as black dot of ostiole, surrounded by a whitish halo. ***Clypeus*** carbonaceous, rudimentary, thick-walled, the ostiolar opening surrounded with black cells. ***Ostioles*** centric, ostiolar canal periphysate. ***Peridium*** 17–30 μm (x̄ = 23 μm, n = 20) wide, tightly attached to the host tissue, with two cell layers, outer layer thick-walled, comprising yellowish brown cells of *textura angularis*, inner layer thin, composed of hyaline cells of *textura angularis*. ***Paraphyses*** 3.7–7.6 μm (x̄ = 5.4 μm, n = 25) wide, wider at the base, longer than the asci, filamentous, septate, constricted at the septa, embedded in gelatinous matrix. ***Asci*** 112–158 × 8.5–13.7 μm (x̄ = 131 × 11 μm, n = 25), 8-spored, unitunicate, cylindrical, short pedicellate, apically rounded, with 3.9–5.6 × 2.5–3.7 μm (x̄ = 5 × 3 μm, n = 18), rectangular to slightly obconic, apical ring, J+ in Melzer’s reagent. ***Ascospores*** 14.5–17.6 × 6–7.4 μm (x̄ = 16.3 × 6.7 μm, n = 30), L/W 2.4, oblong to ellipsoidal, uniseriate, brown, inequilaterally unicellular, apical cell 13–15.5 μm (x̄ = 14.5 μm, n = 30) long, usually with large guttules, brown cell with a mucilaginous sheath covering most of the spore length when mature, with a small, hyaline, rounded, basal cell, 1.3–2.3 μm (x̄ = 1.8 μm, n = 30), lack of germ slit. ***Asexual morph***: Undetermined.

**Figure 8.**
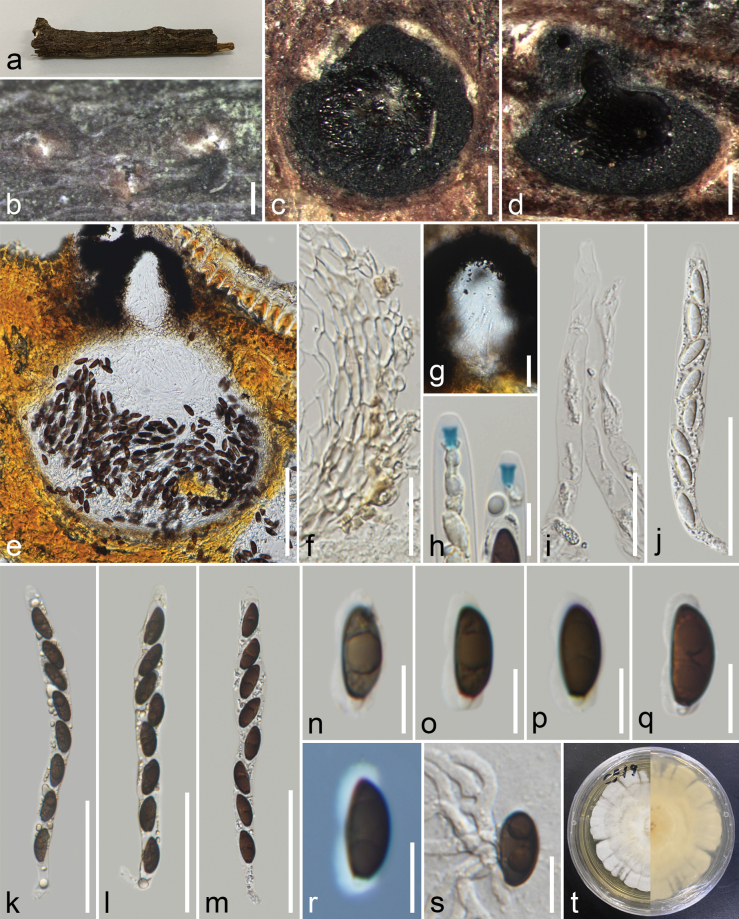
*Occultithecachiangraiensis* (MFLU 24–0414, holotype) **a** host substrate **b** ascomata in host surface **c, d** horizontal and vertical section of ascoma **e** section through ascoma **f** peridium **g** ostiole **h** apical ring stained with melzer’s reagent **i** paraphyses **j–m** immature and mature asci **n–r** ascospores (**r** ascospores show mucilaginous sheath in indian ink) **s** a germinated spore **t** front and reverse view of the colony on PDA. Scale bars: 200 μm (**b**); 100 μm (**c–e**); 20 μm (**f, g, i**); 10 μm (**h, n–s**); 50 μm (**j–m**).

##### Culture characteristics.

Colonies on PDA, reaching 40 mm diam., after 21 days at 25 °C, circular, entire edge, smooth surface, flat, slightly woolly, smooth margin, above ash white from the centre to white at the edge with concentric rings of woolly; from below: light brown at the centre, white at the margin, with ash white mycelium.

##### Material examined.

Thailand, Chiang Rai, Mae Fah Luang District, Mae Salong Nok, on a dead unidentified dicot branch, 16 January 2023, J. Louangphan, CR1–19 (MFLU 24–0414, holotype); ex-type MFLUCC 25–0158.

##### Host.

Unidentified branch (this study).

##### Distribution.

Thailand (this study).

##### Notes.

Based on multi-gene phylogenetic analyses of ITS, LSU, *rpb2*, and *tub2* sequences, *Occultithecachiangraiensis* (MFLU 24–0414) clustered with *O.rosae* (HKAS 102393) and *Clypeosphaeriaoleae* (CPC 36779) with 100% ML and 1.00 BPP support (Fig. 7). *Clypeosphaeriaoleae* was reported only from the asexual morph (Crous et al. 2019); thus, we could not compare the morphology between the species as we could not obtain the asexual morph of our strain, and therefore the link between them cannot be confirmed. Furthermore, *C.oleae* lacks *rpb2* and *tub2* data, which is important to confirm its phylogenetic placement. Morphologically, *Occultithecachiangraiensis* fits the generic concept of *Occultitheca* in having immersed ascomata, short pedicellate asci with a J+, apical ring, a long distance between the ascus apex and the uppermost ascospore, and hyaline basal cells attached to brown ascospores (Rogers and Ju 2003; Samarakoon et al. 2022). Our isolate was compared to *Occultitheca* species as detailed in Table 4. *Occultithecachiangraiensis* differs from *O.rosae* by having smaller ascomata (x̄ = 290 × 324 μm vs. 370 × 385 μm), a lack of a germ slit, and possesses a thicker mucilaginous sheath compared to *O.rosae* (Samarakoon et al. 2022). Our strain differs from *O.ananasi*, which has uniseriate, olive-greenish ascospores becoming 2-seriate in the middle and thin mucilaginous sheath (Tian et al. 2024). *Occultithecachiangraiensis* was also compared to the type species *O.costaricensis* as it lacks molecular data. *Occultithecachiangraiensis* differs by having 1–2 individual ascomata and ascospores with a mucilaginous sheath, while *O.costaricensis* has 2–12 ascomata in a cluster and ascospores without a sheath (Rogers and Ju 2003). Additionally, some *Anthostomella* species have similar characteristics in terms of ascoma, asci, and ascospores with draft cells and lack germ slits, such as *A.clypeata* and *A.clypeoides*, but differ in a short space of the top ascospore and the ascus apex and shape of the sheath compared to *Occultitheca* species (Lu and Hyde 2000; Rogers and Ju 2003). Our strain differs by 6% in the ITS region (30/482, 4 gaps), 2% in LSU (14/745, 4 gaps), 15% in *rpb2* (117/798, no gap), 16% in *tub2* (120/754, 22 gap), and 3% in *tef1* (31/915, 1 gap) sequences compared to *O.rosae* (HKAS 102393). Thus, *Occultithecachiangraiensis* is reported as a new species based on morphology and phylogenetic evidence.

**Table 4.** Synopsis of *Occultitheca* species.

Species	*O.costaricensis*	*O.rosae*	*O.ananasi*	*O.chiangraiensis*
Host	Unidentified decaying wood	*Rosa* sp.	*Ananascomosus*	Unidentified decaying wood
Country	Costa Rica	China	Thailand	Thailand
Ascomata (μm)	400–600	360–385 × 350–420	190–230 × 160–260	220–342 × 228–395
Peridium (μm)	−	18–25	15–20	17–30
Paraphyses (μm)	−	3–6.5	3–5	3.7–7.6
Asci (μm)	185–190 × 10–10.5	90–140 × 11–13	70–90 × 5–10	112–158 × 8.5–13.7
Apical ring (μm)	6 × 3	3.5–4.5 × 2.8–3.2	−	3.9–5.6 × 2.5–3.7
Ascospores (μm)	14.5–23.5 × 7–10.5	16.5–20 × 6.5–8 L/W 2.6	10–12.5 × 3.5–4.5 L/W 2.9	14.5–17.6 × 6–7.4 L/W 2.4
Basal cell (μm)	1.5–4.5	1.5–2.2	−	1.3–2.3
Spore sheath	No	Thin	Thin	One side thick
Germ slit	Straight	Straight	Straight	No
References	Rogers and Ju (2003)	Samarakoon et al. (2022)	Tian et al. (2024)	This study

− Data not available.

## ﻿Discussion

In this study, two novel species, *Diaporthethailandica* and *Occultithecachiangraiensis*, along with a new host record of *D.tulliensis* and a new geographical record of *D.melonis*, are introduced based on morphology and molecular data. This study expands the known diversity of these taxa and highlights the importance of saprobic microfungi in ecological systems.

*Diaporthe* is a species-rich genus with a diverse host range and global distribution (Dissanayake et al. 2017; Phukhamsakda et al. 2020; Hongsanan et al. 2023; Bhunjun et al. 2024b). The species have overlapping morphological traits. There are more than 1200 epithets under *Diaporthe* in Index Fungorum (2024); thus, the boundaries of the species/species complexes within the genus have been revised by several studies. *Diaporthe* was recently restructured into seven sections and 15 species complexes based on molecular analyses. Several *Diaporthe* species have been synonymised under *D.tulliensis* (*D.alangii*, *D.celtidis*, *D.glabrae*, *D.hubeiensis*, *D.morindae*, and *D.tectonae*), and these taxa formed a clade in our trees (including in the backbone tree with 226 taxa; data not shown), similar to Dissanayake et al. (2024). *Diaporthemelonis* (= *D.guangdongensis*) also formed a clade in our trees (including in the backbone tree; data not shown), similar to Dissanayake et al. (2024). The implementation of the markers (ITS, *tef-1*, and *tub*) proved to be phylogenetically informative in this study, resulting in a similar topology as previous studies based on five-marker combinations. The GCPSR analysis was also used to support the novelty of the *Diaporthe* species in this study. Therefore, molecular data and morphological evidence are needed for accurate species identification, thus reinforcing the importance of integrative approaches.

Morphologically, *Occultitheca* is considered an inconspicuous xylarialean and treated as anthostomella-like taxa in terms of having immersed, clypeate ascomata, asci with a J+, apical ring, ascospores with a large brown cell, and a basal hyaline dwarf cell (Daranagama et al. 2018; Samarakoon et al. 2022). Anthostomella-like taxa have now been split into five genera as suspected by Daranagama et al. (2015). These include *Anthocanalis*, *Astrocystis*, *Brunneiperidium*, *Lunatiannulus*, and *Pyriformiascoma*, and they differ in terms of ascomata and asexual morph characters. *Occultitheca* has a distinctive ostiole surrounded by a whitish halo and apiosporous ascospores with a small hyaline dwarf cell at one end and a dark brown, larger cell. There are only three species in *Occultitheca*, and all of them were found as saprobes as sexual morphs from terrestrial ecosystems. *Occultitheca* has a limited distribution and a narrow range of hosts, as only one species was found in Costa Rica (Rogers and Ju 2003), one in China, and one in Thailand (Samarakoon et al. 2022; Tian et al. 2024). Here, we provide a new addition to *Occultitheca* from unidentified decaying wood in Chiang Rai, Thailand. *Occultithecachiangraiensis* was reported only as a sexual morph due to failure to obtain the asexual morphs from cultures. Thus, the link between the sexual and asexual morphs of this genus remains unknown. Due to the uncertainty of asexual and sexual morphologies and the low number of collections, *Occultitheca* has been placed under Xylariales genera *incertae sedis* (Samarakoon et al. 2022; Tian et al. 2024) and has also been considered to be part of Xylariaceae based on phylogenetic analyses (Voglmayr et al. 2022; this study). Expanding the sample collection will improve the representativeness of the genus.

This study provides a vital contribution to our understanding of Sordariomycetes diversity. Introducing new taxa is significant as they contribute to the broader understanding of fungal evolution, taxonomy, and ecology. It also contributes to the growing knowledge about the diversity of fungi associated with woody litter. It emphasises the necessity for continued exploration of fungal biodiversity across various habitats. As global ecosystems undergo rapid changes due to climate shifts and habitat destruction, understanding these dynamics will be crucial for conservation efforts and ecosystem management. Therefore, this study encourages further exploration in understudied substrates and regions, which could unveil additional species and enrich our comprehension of fungal ecology and taxonomy.

## Supplementary Material

XML Treatment for Diaporthe melonis

XML Treatment for Diaporthe thailandica

XML Treatment for Diaporthe tulliensis

XML Treatment for Occultitheca chiangraiensis

## ﻿Additional information

### Author ORCIDs

Chayanard Phukhamsakda https://orcid.org/0000-0002-1033-937X

Kevin D. Hyde https://orcid.org/0000-0002-2191-0762

Milan C. Samarakoon https://orcid.org/0000-0002-4815-125X

Johnny Louangphan https://orcid.org/0000-0003-3845-6145

Kedsara Navasit https://orcid.org/0009-0004-8167-0600

Fatimah Al-Otibi https://orcid.org/0000-0003-3629-5755

### Data availability

All of the data that support the findings of this study are available in the main text.
